# Poverty, urban-rural classification and term infant mortality: a population-based multilevel analysis

**DOI:** 10.1186/s12884-019-2190-1

**Published:** 2019-01-22

**Authors:** Yousra A. Mohamoud, Russell S. Kirby, Deborah B. Ehrenthal

**Affiliations:** 1 Department of Population Health Sciences, School of Medicine and Public Health, University of Wisconsin, WARF Office Building, Room 675A, 610 Walnut St, Madison, WI 53726 USA; 2 Department of Community and Family Health, College of Public Health, University of South Florida, Tampa, FL USA; 3 Department of Obstetrics and Gynecology, School of Medicine and Public Health, University of Wisconsin, Madison, WI USA

**Keywords:** Poverty, Urban, Rural, Term births, Infant mortality, Multilevel analysis

## Abstract

**Background:**

U.S. mortality rate of term infants is higher than most other developed countries. Term infant mortality is associated with exogenous socio-environmental factors. Previous research links low socioeconomic status and rurality with high infant mortality, but does not examine the effect of individual level factors on this association. Separating out the effect of contextual factors from individual level factors has important implications for targeting interventions. Therefore, we aim to estimate the independent effect of poverty and urban-rural classification on term infant mortality.

**Methods:**

We used linked 2013 period cohort birth-infant death files from the National Center for Health Statistics (NCHS). Counties were assigned to low, medium and high poverty groups using US Census Bureau county-level percent of children ≤18 years living in poverty, and were classified based on NCHS urban-rural classification. Bivariate and multilevel logistic regression models were used to estimate odds of term infant death, accounting for individual and county level variables.

**Results:**

There were 2,551,828 term births in 2013, with an overall term mortality of 2.1 per 1000 births. Odds of term infant mortality increased from 1.4 (95% CI: 1.2, 1.6) to 1.8 (95% CI: 1.6, 2.0) comparing births over increasing county poverty to those in the lowest. The associations remained significant in the multivariable model, for highest poverty 1.3 (95% CI: 1.1, 1.5). Similarly, the odds of term infant mortality increased with increasing rurality, from 1.3 (95% CI: 1.2, 1.5) in medium metro counties to 1.7 (95% CI: 1.5, 2.0) in non-core counties compared to large fringe metro counties. However, only rural non-core counties remained statistically associated with increased risk of term infant mortality after adjusting for individual level maternal characteristics.

**Conclusions:**

High poverty and very rural counties remained associated with term infant mortality independent of individual maternal sociodemographic, health and obstetric factors. Interventions should focus on contextual factors such as economic environment and availability of health and social services in addition to individual factors to reduce term infant mortality.

## Background

Despite the decline in infant mortality in recent years, the United States (US) infant mortality rate (IMR) remains higher than most other developed countries. While US IMR for very preterm infants compares favorably to most European rates, the rate for term births, defined as births at 37 weeks gestation or more, is the highest [1]. Surprisingly, there is a dearth of research investigating term infant mortality. One study identified term births as “a period of heterogeneous risk”, found that term deaths tend to occur in the postneonatal period, with accidents, assault and sudden infant death syndrome (SIDS) being the leading causes [2]. Deaths during this period are more likely to be associated with exogenous socio-environmental factors such as housing conditions, infant sleep environment, access to health care, nutrition and social services [3].

The inability of individual level factors, behavioral and psychosocial, to fully explain socioeconomic and racial gradients in birth outcomes has focused attention in recent years on the potential role of more distal factors including the broader macroeconomic, social and geographic contexts in producing adverse birth outcomes [4, 5]. Although difficult to isolate, many studies have shown an increasing risk of preterm birth and low birth weight with increasing contextual socioeconomic disadvantage [5–8]. However, fewer studies have looked at contextual socioeconomic disadvantage and infant mortality. Those that do, tend to adjust for only a minimal set of individual level factors [9, 10]. Similarly, while studies show higher IMR, particularly postneonatal mortality, in rural counties compared to urban counties, none adjust for individual level factors to isolate contextual rural-urban class effects [11, 12].

The purpose of this study, therefore, is to build on previous literature by investigating the association between socio-environmental factors and term infant mortality. More specifically, we aim to estimate the independent effect of county poverty and urban-rural classification on term infant mortality. We take advantage of recent richer large population-based data, and consider a robust set of individual level factors.

## Methods

### Data source

We used data from the 2013 period linked birth/infant death data file obtained from the National Center of Health Statistics (NCHS) to examine infant survival among infants born at term. Period linked infant/death files include all deaths in 2013 whether births occurred in 2012 or 2013 in the numerator and all births in 2013 in the denominator. Federal information processing (FIPS) county codes indicating mother’s county of residence were used to link the birth/infant death data with 2010 model-based small county poverty estimates produced by the U.S. Census Bureau [13] and the 2013 NCHS Urban-Rural Classification scheme for counties which is based on the Office of Management and Budget’s (OMB) February 2013 delineation of metropolitan statistical areas (MSA) and micropolitan statistical areas (derived according to the 2010 OMB standards for defining these areas) and Vintage 2012 post-censal estimates of the resident U.S. population [14]. Our population of interest was all singleton births ≥37 weeks gestation (“term births”). Because several variables of interest including maternal education, prenatal care utilization, maternal smoking and payment source were substantively changed or newly added with the 2003 revision to the U.S. Standard Certificate of Live Birth, we excluded data from the 10 states that had not implemented the 2003 revision by January 1, 2013 (Alabama, Arkansas, Arizona, Connecticut, Hawaii, Maine, Mississippi, New Jersey, Rhode Island, and West Virginia) [15]. We further restricted the analysis to records within a plausible range of gestational age (37–44 weeks) and birthweight for term gestation (1000-6000 g). Finally, we excluded women with missing data on any covariate of interest to conduct complete case analysis. Ten percent of observations had missing information on a covariate of interest mainly on prenatal care initiation (4%), tobacco use (5%) and payer source (6%). Our final analytic sample was 2,551,828 births in 2778 counties.

At the county level, exposure of interest, poverty was defined as percent of children living within a county at or below the poverty line obtained from the U.S. Census Bureau’s American Community Survey [13]. Children are at a greater risk for poverty, so in addition to being highly correlated with other poverty measures, it is more sensitive to changes in poverty levels [16] and more relevant to the lives of women with newborns. Counties were assigned to one of three poverty groups: < 10% (low poverty), 10.0–19.9% (medium poverty), and ≥ 20.0% (high poverty), as have previously been defined [17–19]. County urban-rural classification followed the NCHS urban-rural classification with six levels: four metropolitan (large central metro, large fringe metro, medium metro, and small metro) and two non-metropolitan (micropolitan and noncore) [14]. According to NCHS, metropolitan counties include: Large central metro counties defined as MSA of 1 million population that: 1) contain the entire population of the largest principal city of the MSA, or 2) are completely contained within the largest principal city of the MSA, or 3) contain at least 250,000 residents of any principal city in the MSA. Large fringe metro counties defined as MSA of 1 million or more population that do not qualify as large central Medium metro counties in MSA of 250,000–999,999 population. While small metro counties are counties in MSAs of population size less than 250,000. Nonmetropolitan counties include: Micropolitan counties in a micropolitan statistical area; and Noncore counties that are not in micropolitan statistical areas [14].

Individual level characteristics came from birth records and were selected based on clinical relevance and association with infant mortality. Sociodemographic variables included: maternal age (years), maternal education, marital status, and maternal race/ethnicity. Maternal health and obstetric characteristics included: gestational age (weeks), number of previous live births, infant sex, month prenatal care began, tobacco use during pregnancy, history of chronic diabetes, chronic hypertension or pregnancy-related hypertension. We adjusted for medical payer source (Medicaid yes/no) as a proxy for individual income to further isolate the effect of county poverty from individual socioeconomic status. Medicaid provides health coverage to eligible low income individuals, therefore having Medicaid as payer source can be a proxy for low individual socioeconomic status, compared private or self-pay.

### Statistical analysis

Bivariate logistic regressions were conducted to estimate the overall relation of the main exposure (county poverty), county and individual level covariates on term infant mortality. Multilevel logistic regression modelling was conducted to determine the association between poverty, urban-rural classification and term infant mortality, and whether this association was mediated by individual level sociodemographic, maternal health and obstetric characteristics. This approach allows us to estimate not only the fixed effects of context and individual factors, but also the random effect of geographic variation on term infant mortality. The models allowed the intercept for each county to include a random error component relaxing the conditional independence assumptions for birth outcomes of mothers residing in the same state and county. Intraclass correlation coefficient (ICC) was assessed.

We ran models separately for county variables (poverty and urban-rural classification), and individual level sociodemographic, maternal health and obstetric characteristics variables. Finally, we ran a fully-adjusted model including both county and individual level variables to estimate the independent effect of contextual county variables in the final model. To investigate if the effect of poverty by urban-rural classification we tested for interactions between the two county level variables. Odds ratios and 95% CIs for each of the models are presented. We used directed acyclic graphs (DAG) to guide our analysis by describing a plausible causal model for county poverty and urban-rural classification as independent risk factors for term infant mortality. To assess the sensitivity of findings to spatial correlation between counties, we re-ran the final fully adjusted model including longitude and latitude co-ordinates of county centroids. All analyses were completed using Stata/IC 14.2 (Stata Corp, College Station, TX).

## Results

### Poverty, urban-rural classification and term infant mortality

**Fig. 1:**
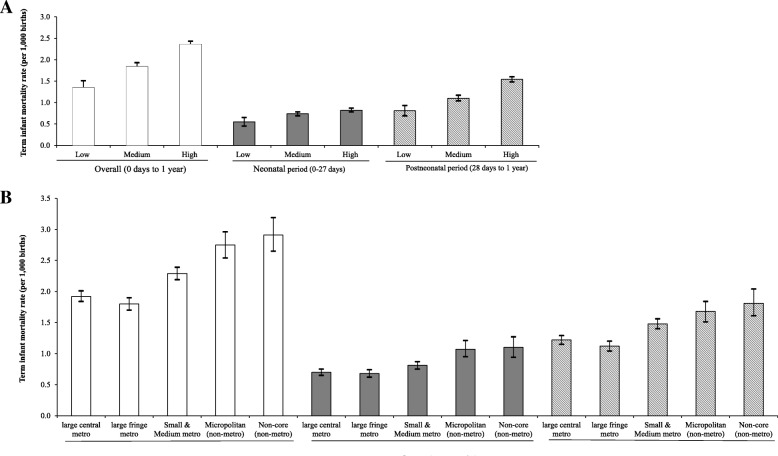
Distribution of term infant mortality rates overall, and in neonatal and postneonatal periods across **a** county poverty levels and **b** urban-rural classifications in the US 2013

Term infant mortality rate in this cohort was 2.1 per 1000 births. Figure 1 shows the distribution of term infant mortality overall and in the neonatal and postneonatal periods by county poverty and urban-rural classification. Term infant mortality rate in high poverty counties was almost twice as much as in low poverty counties. Compared to low poverty counties, the neonatal mortality rate was 38% higher and the postneonatal rate 47% higher in high poverty (*p* < 0.001). There was an increasing trend in overall term infant mortality with increasing rurality, driven by higher postneonatal mortality rates with increasing rurality.

### Poverty, urban-rural classification and individual level factors

Table 1 outlines the distribution of county and individual level characteristics of term births across county poverty levels. Two-thirds of term births occurred among mothers residing in high poverty counties. High poverty counties saw the highest births in non-metropolitan areas, the highest teen pregnancy rates and the highest birth rates to mothers with high school education or less. Mothers residing in high poverty counties were less likely to be married and more likely to be NH-black or Hispanic compared to those residing in medium or low poverty counties. These mothers were less likely to be nulliparous and more likely to have had no prenatal care or to have accessed prenatal care in the third trimester.

**Table 1:** Distribution of maternal county of residence urban-rural classification, sociodemographic, health and obstetric characteristics of term births by county poverty^a^, US 2013

	Low Poverty		Medium Poverty		High Poverty	
	N	(%)	N	(%)	N	(%)
	227,760	7.6	979,088	32.5	1,804,312	59.9
Contextual County Characteristics						
County urban-rural classification						
Large central metro	9024	4.0	207,356	21.2	806,000	44.7
Large fringe metro	206,349	90.6	379,031	38.7	125,059	6.9
Medium metro	7581	3.3	195,098	19.9	410,336	22.7
Small metro	2144	0.9	88,796	9.1	173,863	9.6
Micropolitan (non-metro)	1498	0.7	70,780	7.2	172,164	9.5
Non-core (non-metro)	1164	0.5	38,027	3.9	116,890	6.5
Maternal Individual Characteristics						
Maternal age (years)						
≤ 19	6947	3.1	51,062	5.2	149,170	8.3
20–24	28,213	12.4	193,690	19.8	464,937	25.8
25–29	61,745	27.1	290,656	29.7	518,092	28.7
30–34	81,815	35.9	285,546	29.2	433,099	24.0
35–39	40,034	17.6	128,818	13.2	193,808	10.7
≥ 40	9006	4.0	29,316	3.0	45,206	2.5
Maternal education						
High school or less	51,938	22.8	320,593	32.7	822,059	45.6
Some college	54,431	23.9	294,302	30.1	522,084	28.9
Bachelor’s or more	118,188	51.9	346,468	35.4	438,619	24.3
Marital status (Yes)	172,872	75.9	662,214	67.6	984,766	54.6
Maternal race/ethnicity						
Non-Hispanic White	149,335	65.6	631,496	64.5	869,560	48.2
Non-Hispanic Black	19,253	8.5	89,074	9.1	306,724	17.0
Hispanic	34,684	15.2	169,091	17.3	500,517	27.7
Non-Hispanic Other	23,485	10.3	79,960	8.2	116,621	6.5
Gestational age (weeks)						
37	16,830	7.4	72,036	7.4	148,629	8.2
38	38,402	16.9	162,013	16.6	327,337	18.1
39	96,639	42.2	406,034	41.5	768,118	42.6
40	57,841	25.4	250,770	25.6	431,416	23.9
≥ 41	18,048	7.9	88,235	9.0	128,812	7.1
Parity						
0	93,972	41.3	394,077	40.3	714,573	39.6
1–2	114,986	50.5	477,582	48.8	860,991	47.7
≥ 3	18,215	8.0	102,909	10.5	219,841	12.2
Infant sex (Female)	112,249	49.3	479,520	49.0	885,644	49.1
Month prenatal care began						
First trimester	171,993	75.5	733,075	74.9	1,244,999	69.0
Second trimester	33,132	14.6	166,895	17.1	367,761	20.4
Third trimester	8204	3.6	36,807	3.8	92,294	5.1
No prenatal care	1163	0.5	6731	0.7	26,439	1.5
Tobacco use during pregnancy (Yes)	10,852	4.8	79,571	8.1	149,346	8.3
Chronic diabetes (Yes)	12,152	5.3	58,522	6.0	97,500	5.4
Chronic hypertension (Yes)	2635	1.2	12,035	1.2	23,222	1.3
Pregnancy associated hypertension (Yes)	7495	3.3	39,942	4.1	71,349	4.0
Medicaid (Yes)	47,823	21.0	324,144	33.1	897,407	49.7

^a^low poverty (< 10%), medium poverty (10.0–19.9%), high poverty (≥20%)

### Multivariable analysis

Table 2 (Model 1) shows the result of bivariate logistic regressions estimating the odds of term infant mortality. The unadjusted odds of term infant mortality increased with increasing poverty, with the births in medium poverty counties having 1.4 times (95% CI: 1.2, 1.7), and births in high poverty counties having 1.8 times (95% CI: 1.6, 2.0), the odds of infant mortality than infants whose mothers live in low poverty counties. Similarly, the risk of term infant mortality increased with increasing rurality, with the highest odds observed in non-core (non-metro) areas 1.7 (95% CI: 1.5, 2.0) compared to large fringe metropolitan areas. The odds of term infant mortality was 2.5 (95% CI: 2.3, 2.7) to mothers with a high school education or less compared to mothers with a bachelor’s degree or more. Term mortality among NH-blacks was 1.6 (95% CI: 1.5, 1.7) times that of NH-whites. Mothers with greater than three previous live births had 1.8 (95% CI: 1.7, 2.0) times higher odds of term infant mortality compared to nulliparous mothers. Births paid by Medicaid had 2.0 (95% CI: 1.9, 2.1) greater odds of term infant mortality compared to births paid through a private source.

**Table 2:** Bivariate and multivariate logistic regression estimation of odds of term infant mortality by contextual county characteristics and individual maternal sociodemographic, health and obstetric characteristics in the US, 2013

	Model 1		Model 2		Model 3		Model 4	
	Bivariate		County variables		Individual variables		County + individual variables	
	OR	95%CI	aOR	95%CI	aOR	95%CI	aOR	95%CI
Contextual County Characteristics								
Poverty^≠^								
Low	Referent		Referent				Referent	
Medium	1.41***	(1.19, 1.66)	1.39**	(1.17, 1.64)	–	–	1.16	(0.99, 1.36)
High	1.84***	(1.56, 2.18)	1.90***	(1.60, 2.27)	–	–	1.31***	(1.11, 1.54)
County Urban/Rural Scheme								
Large central metro	1.07	(0.93, 1.22)	0.85*	(0.75, 0.96)	–	–	0.98	(0.9, 1.1)
Large fringe metro	Referent		Referent				Referent	
Medium metro	1.30***	(1.18, 1.46)	1.02	(0.91, 1.15)	–	–	1.05	(0.95, 1.17)
Small metro	1.23***	(1.08, 1.41)	0.97	(0.84, 1.10)	–	–	0.95	(0.83, 1.08)
Micropolitan (non-metro) metro	1.55***	(1.37, 1.76)	1.19**	(1.04, 1.35)	–	–	1.10	(0.97, 1.24)
Non-core (non-metro)	1.72***	(1.51, 1.97)	1.31***	(1.14, 1.50)	–	–	1.16*	(1.02, 1.33)
Maternal Individual Characteristics								
Gestational age (weeks)								
37	2.30***	(2.13, 2.48)	–	–	2.12***	(1.95, 2.31)	2.12***	(1.95, 2.31)
38	1.43***	(1.33, 1.53)	–	–	1.38***	(1.28,1.49)	1.38***	(1.28, 1.49)
39	Referent				Referent		Referent	
40	0.82***	(0.76, 0.88)	–	–	0.85***	(0.78, 0.92)	0.86***	(0.79, 0.93)
≥ 41	0.86**	(0.77, 0.96)	–	–	0.92	(0.81, 1.04)	0.93	(0.82, 1.05)
Maternal age (years)								
15–19	1.62***	(1.48, 1.77)	–	–	1.50***	(1.34, 1.68)	1.47***	(1.31, 1.65)
20–24	1.39***	(1.30, 1.48)	–	–	1.19***	(1.10, 1.29)	1.18***	(1.09, 1.27)
25–29	Referent				Referent		Referent	
30–34	0.74***	(0.69, 0.80)	–	–	0.78***	(0.71, 0.85)	0.79***	(0.72, 0.86)
35–39	0.76***	(0.69, 0.84)	–	–	0.83***	(0.74, 0.92)	0.84**	(0.75, 0.94)
≥ 40	1.25**	(1.08, 1.44)	–	–	1.29**	(1.10, 1.52)	1.33**	(1.12, 1.56)
Maternal education								
High school or less	2.47***	(2.31, 2.65)	–	–	1.30***	(1.18, 1.44)	1.27***	(1.15, 1.40)
Some college/AA	1.81***	(1.68, 1.96)	–	–	1.19***	(1.08, 1.31)	1.17**	(1.06, 1.28)
Bachelor’s or more	Referent				Referent		Referent	
Maternal race/ethnicity			–	–				
Non-Hispanic White	Referent				Referent		Referent	
Non-Hispanic Black	1.63***	(1.53, 1.74)	–	–	1.19***	(1.10, 1.29)	1.20***	(1.11, 1.31)
Hispanic	0.84***	(0.78, 0.89)	–	–	0.68***	(0.62, 0.73)	0.68***	(0.63, 0.74)
Non-Hispanic Other	0.75***	(0.67, 0.84)	–	–	0.87*	(0.76, 0.99)	0.89	(0.78, 1.01)
Marital status								
No	Referent				Referent		Referent	
Yes	0.53***	(0.51, 0.56)	–	–	0.83***	(0.78, 0.89)	0.83***	(0.78, 0.89)
Number of previous live births								
0	Referent				Referent		Referent	
1–2	1.22***	(1.15, 1.29)	–	–	1.32***	(1.24, 1.42)	1.31***	(1.23, 1.41)
≥ 3	1.83***	(1.70, 1.97)	–	–	1.81***	(1.65, 2.00)	1.78***	(1.61, 1.96)
Infant sex								
Male	Referent				Referent		Referent	
Female	0.84***	(0.80, 0.88)	–	–	0.84***	(0.80, 0.89)	0.84***	(0.80, 0.89)
Month prenatal care began								
First trimester	Referent				Referent		Referent	
Second trimester	1.55***	(1.46, 1.65)	–	–	1.25***	(1.16, 1.34)	1.24***	(1.16, 1.33)
Third trimester	2.23***	(2.04, 2.44)	–	–	1.71***	(1.55, 1.90)	1.70***	(1.54, 1.89)
No prenatal care	3.71***	(3.25, 4.23)	–	–	2.12***	(1.80, 2.53)	2.09***	(1.76, 2.49)
Tobacco use during pregnancy								
No	Referent				Referent		Referent	
Yes	2.59***	(2.43, 2.76)	–	–	1.79***	(1.65, 1.94)	1.77***	(1.63, 1.91)
Chronic hypertension								
No	Referent				Referent		Referent	
Yes	1.64***	(1.38, 1.94)	–	–	1.32**	(1.07, 1.62)	1.30*	(1.06, 1.60)
Chronic diabetes								
No	Referent				Referent		Referent	
Yes	1.06	(0.95, 1.18)	–	–	1.02	(0.90, 1.15)	1.02	(0 .90, 1.15)
Pregnancy associated hypertension								
No	Referent				Referent		Referent	
Yes	1.18**	(1.05, 1.32)	–	–	1.04	(0.91, 1.19)	1.03	(0.90, 1.18)
Medicaid								
No	Referent				Referent		Referent	
Yes	1.95***	(1.85, 2.06)	–	–	1.20***	(1.11, 1.28)	1.18***	(1.10, 1.27)
*N*	–		2,551,828		2,551,828		2,551,828	
*ICC*	–		0.015***		–		0.006***	

* *p* < 0.05, ** *p* < 0.01, *** p < 0.001, ^≠^low poverty (< 10%), medium poverty (10.0–19.9%), high poverty (≥20%)

Table 2 (Models 2–4), presents the multivariable logistic regression results. In the county variables only model (Model 2), adjusting for county urban-rural classification did not change the association of poverty with infant survival. On the other hand, adjusting for county poverty attenuated the association of urban-rural classification on term infant mortality, such that they were only significant when comparing non-metro rural areas (micropolitan: 1.2, 95% CI: 1.0, 1.4, and non-core: 1.3, 95% CI: 1.1, 1.5) with large fringe metro areas. Model 3 adjusts for maternal individual variables only. In this model, gestational age continued to have an effect on the risk of term infant mortality with the greatest risk observed comparing births at 37 and 38 weeks to births at 39 weeks gestation. The U-shaped effect of maternal age associated with infant mortality was still observed with term infant mortality. Though somewhat attenuated, low maternal education, prenatal smoking, and late access or no prenatal care as well as being on Medicaid continued to be risk factors of term infant mortality in this model.

Model 4 adjusts for individual sociodemographic, maternal health and obstetric characteristics to isolate the effect of poverty and urban-rural classification on term infant mortality, our main objective of interest. In this model only high poverty counties continued to have a significant, though attenuated effect, with the odds of term infant mortality among births in being 1.3 times greater (95% CI: 1.1, 1.5) than those in counties with low poverty. After adjusting for individual level maternal characteristics and county poverty, an association was observed only in the most rural non-core counties compared to large fringe metro counties (OR 1.2, 95% CI:1.0,1.3), *p* = 0.03. Adjusting for county level contextual variables did not change the association between individual level maternal characteristics and infant mortality.

The multilevel approach used in our final model (Model 4) allowed us to estimate the random effect of geographic variation on term infant mortality. Compared to within county heterogeneity, the additional contribution of between-county heterogeneity to the overall variance was very small. Across models the ICC ranged from 0.015 to 0.008, suggesting that individual level heterogeneity played a more important role in explaining the variability in term infant mortality than geographic county level heterogeneity. Though the ICC estimate was very modest, the likelihood ratio test, testing if the value of (rho, ICC estimate) is statistically significantly different from zero was significant *p* < 0.001 in all three models.

As sensitivity analyses, we re-ran our final model this time adjusting for county centroids to account for potential residual spatial autocorrelation. Our findings remained stable, with no change in the interpretation of our results. We also tested for potential interaction between county poverty and urban/rural classifications but found no evidence of interaction (*p* = 0.49). Finally, we re-ran our final model using the whole data (plurals and singleton births) this time adjusting for plurality and found very small differences in our effect estimates from Model 4 and no change in the interpretation of our results.

## Discussion

**Fig. 2:**
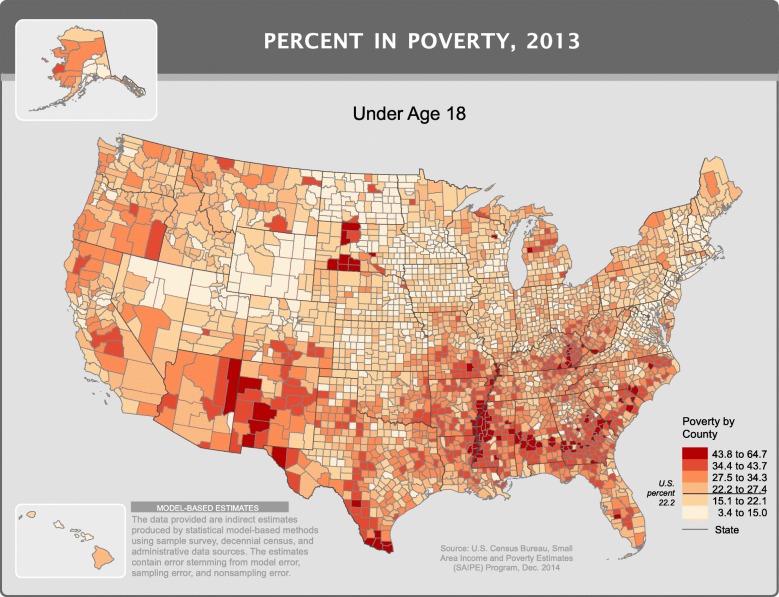
Distribution of children under 18 years living in poverty across counties in the United States in 2013. Source: U.S. Census Bureau, Small Area Income and Poverty Estimates (SAIPE) Program, December 2014 (Figure used with permission from source)

Focusing on an understudied subpopulation of births with relatively higher mortality rates in international comparisons, we estimated the association of poverty and urban-rural classification on the risk of term infant mortality. Increasing poverty was directly associated with odds of term infant mortality, ranging from 1.4 to 1.8-fold higher in medium and high poverty counties when compared to low poverty counties. Although no previous studies specifically focused on term infant mortality and poverty, our results are consistent with findings in studies examining the association of poverty and *overall* infant mortality in the US. Higher infant mortality among low socioeconomic groups has been recognized as a societal problem in the US for 140 years [20, 21]. Still, more than 1 in 4 women giving birth in the US live in counties where more than 20% of children live below the poverty level [22]. Figure 2 was produced by the US Census bureau highlighting the distribution of poverty for children less than 18 years across counties in the United States [23].

Results from our final model (Model 4), show that though attenuated the observed gradient of poverty on term infant mortality persisted after adjusting for sociodemographic, maternal health and obstetric characteristics. High poverty counties had 1.3 (95%CI: 1.1, 1.5) times greater odds of mortality compared to low poverty counties, while 1.2 (95% CI: 0.9, 1.4) greater odds in medium poverty counties. This suggests that geographic resources play a more important role in very high poverty areas than in medium poverty areas where individual behavior and resources seem to explain most of the association. A study analyzing a sample of nearly 13,000 births found that household income matters for infant mortality, especially at very low income levels and even when controlling for a rich set of covariates [9]. While no studies specifically investigated term infant mortality, a study investigating US excess IMR during the *postneonatal period*, used county level median income adjusted for maternal age, education, marital status and race, found that mortality during this period is driven almost entirely by excess infant deaths among mothers of low socioeconomic status [10].

Although we found an effect of county urban-rural classification on term infant mortality in bivariate analyses, this effect was strongly attenuated after adjusting for county poverty. In the final multilevel model (Model 4), only very rural areas remained significantly associated with survival after adjusting for individual level maternal characteristics (OR: 1.2, 95% CI: 1.0, 1.3). This suggests that urban-rural differences in the risk of term infant mortality may act through area level socioeconomic differences and individual factors. Several studies have shown that rural children are more likely to live in poverty, have unmet needs, and rely on Medicaid for their health care [24–27]. The increased risk of term infant mortality in very rural areas suggest a geographic association between rurality and infant mortality perhaps relating to lack of access to health services.

Overall these findings suggest that while individual behavioral factors (e.g. smoking during pregnancy), biological factors (maternal age, medical conditions) and individual resources (education, Medicaid, month prenatal care initiated) are potential mediators between poverty and term infant mortality, poverty and rurality are independently associated with term infant survival. County poverty and rurality may impact term infant survival through their association with access to safe housing, nutrition, social support, and increased exposure to pollution, violence and stress [20, 28–30]. These findings support the importance of policies to reduce poverty [20, 21, 28–30] and are consistent with findings from a study found that a dollar increase in the minimum wage above the federal level was associated with a 4% decrease in postneonatal mortality [31]. In research contrasting infant mortality in Sweden to the US, authors attributed the low IMR in Sweden primarily to the country’s efforts to eliminate poverty and social class differences (for e.g. welfare programs) and secondarily to its health services [20]. This is particularly relevant to term birth mortality, given a recent estimate that 47% of the higher IMR in the U.S. compared to Sweden is among term births [1].

The multilevel approach we used enables the evaluation and quantification of heterogeneity of the outcome across geographic areas. When minimal geographic heterogeneity exists, this implies high area-level correlation. Our findings remained unchanged even after adjusting for county centroids. When individuals within the population are similar and vary little with respect to health, this provides optimal conditions for regional prevention strategies [32, 33]. Likewise, health outcomes such as term infant mortality, which can be significantly influenced by behavior related factors (e.g., smoking, receipt of prenatal care), may be less influenced by area-level factors than diseases with long natural history such as atherosclerotic disorders.

Strengths of our study include using a large population-based dataset covering most 2013 term births occurring in the US and linked it with county level measures of child poverty to obtain sufficiently powered and reliable estimates. Our measure of poverty was chosen as it remains the official national poverty statistic and has the most salience in policy-making. Despite the large sample size, our analysis was limited to the 41 states and the District of Columbia that had fully adopted the 2003 revision of the birth certificate by 2013. While these data represent 90% of all 2013 US births, it is unclear whether the results are generalizable for the country as a whole [15]. Additionally, we had to exclude approximately 10% of our sample due to missing data on covariates of interest. Analysis of the missing information patterns across county level exposures shows that most missing observations were among births in high poverty and large urban metro counties. This suggests that we might be underestimating the association between county poverty and term infant mortality. Furthermore, most missing observations came from three states Michigan, Virginia and Georgia, which creates additional concerns about generalizability. Though we adjusted for a robust set of variables we were limited to the data available on the birth certificate there might be residual effects due to unmeasured confounding at the individual or county level.

Social and economic characteristics vary within counties and observations at this geographic scale may dilute the effect of poverty on infant mortality. Furthermore, Census data might not accurately characterize the demographic context of study subjects because of the requirement of residential address and because of the undercount, which chiefly affects poor people and people of color [34]. However, undercount and missing residential address would most likely dilute the association between term infant mortality and poverty, since it produces conservative estimates of the number and hence proportion of poor persons and people of color. There are several reasons for choosing county as our unit of measurement. Most importantly, US counties are stable sociopolitical and geographic entities, which provide an appropriate socioeconomic, political and community context within which many social and public health policies are formulated. Therefore, using a county-based approach allows for organized implementation of healthcare policies and interventions [33, 35, 36].

## Conclusions

This study addresses a gap in the literature by focusing on factors associated with infant mortality in an understudied sub-population of births with exceptionally high mortality. Though our analyses cannot be strictly interpreted as a mediation analysis, the finding of a mortality gradient with poverty and rurality of a county among term infants emphasizes the need for further research into the underlying mechanisms by which county poverty and rurality impacts term infant survival, for example, through an analysis of cause-specific term infant mortality. Regardless, our results suggest at the continued need for interventions focused on improving the social determinants of health, including a focus on contextual factors such as neighborhood deprivation, housing and residential stability, safety and food security in addition to individual factors such as reducing teen pregnancy and encouraging higher educational attainment among women.

## Abbreviations

DAG: Directed Acyclic Graphs
FIPS: Federal Information Processing
IMR: Infant Mortality Rate
MSA: Metropolitan Statistical Areas
NCHS: National Center of Health Statistics
OMB: Office of Management and Budgets
SIDS: Sudden Infant Death Syndrome
US: United States
